# *In vivo* high-resolution magic angle spinning magnetic resonance spectroscopy of *Drosophila melanogaster* at 14.1 T shows trauma in aging and in innate immune-deficiency is linked to reduced insulin signaling

**DOI:** 10.3892/ijmm_00000450

**Published:** 2010-08-01

**Authors:** VALERIA RIGHI, YIORGOS APIDIANAKIS, DIONYSSIOS MINTZOPOULOS, LOUKAS ASTRAKAS, LAURENCE G. RAHME, A. ARIA TZIKA

**Affiliations:** 1 NMR Surgical Laboratory, Department of Surgery, Massachusetts General Hospital and Shriners Burn Institute, Harvard Medical School;; 2 Athinoula A. Martinos Center of Biomedical Imaging, Department of Radiology, Massachusetts General Hospital;; 3 Molecular Surgery Laboratory, Department of Surgery, Massachusetts General Hospital, Boston, MA 02114, USA

**Keywords:** magnetic resonance spectroscopy, high resolution magic angle spinning, total through bond correlation spectroscopy, *Drosophila melanogaster*, biomarkers, immunity, insulin signaling, obesity, aging

## Abstract

*In vivo* magnetic resonance spectroscopy (MRS), a non-destructive biochemical tool for investigating live organisms, has yet to be used in the fruit fly *Drosophila melanogaster*, a useful model organism for investigating genetics and physiology. We developed and implemented a high-resolution magic-angle-spinning (HRMAS) MRS method to investigate live *Drosophila* at 14.1 T. We demonstrated, for the first time, the feasibility of using HRMAS MRS for molecular characterization of *Drosophila* with a conventional MR spectrometer equipped with an HRMAS probe. We showed that the metabolic HRMAS MRS profiles of injured, aged wild-type *(wt)* flies and of immune deficient *(imd)* flies were more similar to *chico* flies mutated at the *chico* gene in the insulin signaling pathway, which is analogous to insulin receptor substrate 1–4 (IRS1–4) in mammals and less to those of adipokinetic hormone receptor *(akhr)* mutant flies, which have an obese phenotype. We thus provide evidence for the hypothesis that trauma in aging and in innate immune-deficiency is linked to insulin signaling. This link may explain the mitochondrial dysfunction that accompanies insulin resistance and muscle wasting that occurs in trauma, aging and immune system deficiencies, leading to higher susceptibility to infection. Our approach advances the development of novel *in vivo* non-destructive research approaches in *Drosophila*, suggests biomarkers for investigation of biomedical paradigms, and thus may contribute to novel therapeutic development.

## Introduction

High-resolution magic angle spinning (HRMAS) proton magnetic resonance spectroscopy (^1^H-MRS) is a novel nondestructive technique that substantially improves spectral line-widths and allows high-resolution spectra to be obtained from intact cells, cell culture tissues (1,2), and unprocessed tissue (3–7). HRMAS ^1^H-MRS has enabled us to investigate relationships between metabolites and cellular processes. For example, choline (Cho)-containing compounds involved in phospholipids metabolism and lipids, such as triglycerides, that are involved in apoptosis have been studied (8–11). Although 1D HRMAS ^1^H-MRS techniques can reveal a number of large well-resolved NMR signals, the advent of 2D NMR (12) spectroscopy enabled HRMAS ^1^H-MRS, which provides more detailed analysis and unequivocal assignment of overlapping resonances of biologically important meta-bolites in intact tissue samples (7,8,13–15). It has recently been suggested that an optimized adiabatic TOBSY (Total through Bond correlation SpectroscopY) solid-state NMR pulse sequence for two-dimensional ^1^H-^1^H homonuclear scalar-coupling mixing may reduce acquisition time and improve signal-to-noise (SNR) gain relative to its liquid-state analogue TOCSY (TOtal Correlation SpectroscopY) (16). Nevertheless, to date, HRMAS ^1^H-MRS has only been performed *ex vivo*.

*In vivo* studies of ^1^H MRS combined with *ex vivo* HRMAS ^1^H MRS have revealed intramyocellular lipids (IMCLs) in rodents (11,17), while other *ex vivo* HRMAS ^1^H MRS studies have focused on lipid metabolism (18). Szczepaniak *et al* demonstrated that IMCL stores could be quantified accurately in a clinical setting by ^1^H NMR spectroscopy *in vivo*(19). Van der Graaf *et al* reported recently that ^1^H MRS in humans shows an inverse correlation between IMCL content in human calf muscle and local glycogen synthesis rate (20). Another previous study has outlined the importance of these resonances as biomarkers of insulin resistance in type-2 diabetes patients and their offspring (21). IMCL content in the soleus muscle was found to be increased in insulin-resistant elderly patients, providing support for the hypothesis that an age-associated decline in mitochondrial function contributes to insulin resistance (22).

We anticipated that *in vivo* HRMAS ^1^H-MRS might be a useful tool in *Drosophila* since *in vitro* MRS has been demonstrated to show metabolic effects of hypoxia (23) and temperature stress (24) in flies. *Drosophila* is a useful model organism for investigating genetics and physiology as well as metabolism (25). Yet, with the exception of the recent study of the feasibility of *in vivo* MRI in fruit flies (26), *in vivo* MRS studies in *Drosophila* have not been reported. Thus, we set out to develop an *in vivo* HRMAS ^1^H-MRS methodology in *Drosophila* for the first time, with the aim of advancing non-destructive *in vivo* research approaches in *Drosophila*. Such research would be particularly useful for assessing biomarkers of pathophysiology with the long-term goal of providing critical information that may direct novel therapeutic development.

We applied our newly developed *in vivo* HRMAS ^1^HMRS methodology in *Drosophila* to a study designed to test the hypothesis that trauma and innate immunity is linked to reduced insulin signaling, a phylogenetically conserved pathway for regulation of glucose and lipid metabolism (27,28). This hypothesis was tested in traumatized aged flies as well as in flies with a disorder of the innate immune system using as controls *Drosophila* adipokinetic hormone receptor *(akhr)* mutant flies and *chico* mutant flies with mutations in insulin receptor substrate (IRS), a *Drosophila* homolog of vertebrate IRS1–4, who overexpress triglycerides. Innate immunity deficient *(imd)* flies were used to model immuno-compromised patients (i.e., due to old age, AIDS or cancer patients) whose pathophysiology, such as mitochondrial dysfunction, muscle wasting and increased susceptibility to infection, may be linked to insulin resistance. Lipid meta-bolites were measured in aged *imd* flies subjected to traumatic injury, as well as in *akhr* knockout and *chico* flies with a triglyceride overexpression phenotype, and compared to values obtained in young and aged wild-type *(wt)* control flies, young *imd* flies, and *akhr* or *chico* genetic control flies.

## Materials and methods

### Drosophila flies

We used *Drosophila melanogaster wt* Oregon-R and innate imunity mutants *(imd)* flies (29). To test our hypothesis we used the following flies as controls: a) *akhr^null^* mutants with obese phenotype, and their genetic control strain flies (*akhr^rev^*) (30,31); and b) *chico^1/2^* flies, bearing two mutated alleles of the *chico* gene, a *Drosophila* homolog of vertebrate insulin receptor substrate 1–4 (IRS1–4) and their genetic control *chico^1/+^* flies (32). All flies were male. Young flies were 5–8-day-old, and old flies were 30–33-day-old. Each group consisted of 7 flies. Experiments were performed on: a) control healthy, intact flies; and b) traumatized flies, injured 24 h prior to HRMAS MRS measurement with thoracic non-lethal, needle puncture (33,34). Prior to insertion in the spectrometer, each fly was anesthetized by placing it on ice for <1 min. Flies were kept at 4°C while in the spectrometer. All traumatized flies were placed in the spectrometer 24 h after trauma and special care was taken to avoid inflicting further injury during moving in and out of the rotor. The flies weighed 0.7–1 mg at the time of experiment. All flies survived the ^1^H HRMAS MR spectroscopy experiment, which was completed in ∼45 min per fly.

### In vivo HRMAS ^1^H MR spectroscopy

**Figure 1:**
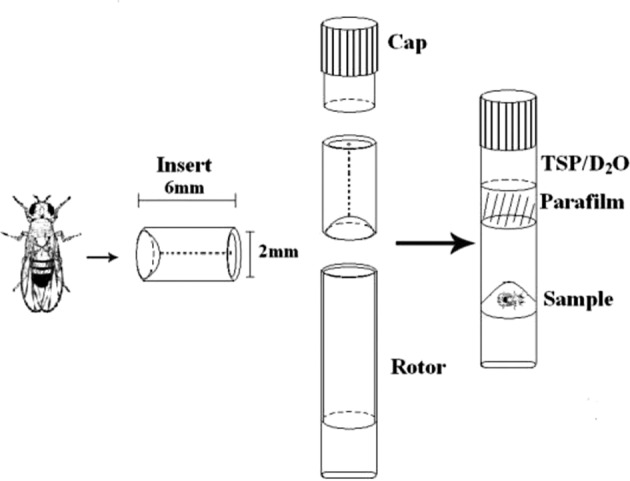
Experimental set up of *in vivo* HRMAS ^1^H MRS for the investigation of live *Drosophila* at 14.1 T. External standard trimethylsilyl-propionic-2,2,3,3-d4 acid (TSP).

All HRMAS ^1^H MRS experiments were performed on a wide-bore Bruker Bio-Spin Avance NMR spectrometer (600.13 MHz) using a 4-mm triple resonance (^1^H, ^13^C, ^2^H) HRMAS probe (Bruker). The flies were placed into a zirconium oxide (ZrO_2_) rotor tube (4-mm diameter, 50 *μ*l), and 8 *μ*l of an external standard trimethylsilylpropionic-2,2,3,3-d4 acid (TSP, Mw=172, d=0.00 ppm, 50 mM in D_2_O) solution were added that functioned as a reference for both resonance chemical shift and quantification. Each fly was placed in the rotor using the insert and the insert was closed with a screw and covered with parafilm to prevent the contact between the fly and the TSP/D_2_O solution (Fig. 1). The samples were secured and tightened in the rotors with a top cap (Bruker). The HRMAS ^1^H MRS was performed at 4°C with 2 kHz MAS.

One-dimensional (1D) water-suppressed spin-echo Carr-Purcell-Meiboom-Gill (CPMG) pulse sequence [90°-(τ-180°-τ)_n_-acquisition] (35) was performed on single flies. CPMG is a methodological improvement of particular interest in developing 1D HRMAS for intact tissue samples *ex vivo*, in order to suppress broad signals that distort the linear baseline in typical Free Induction Decay (FID) spectra. Thus, the CPMG proton NMR spectra are free from the broad ‘rolling’ component that contributes to the baseline of the simple FID spectra. The CPMG sequence has also been applied to 2D sequences for the same reason. Additional parameters for the CPMG sequence included an inter-pulse delay of τ = 2π/ω_r_ = 250 *μ*sec, a total spin-echo delay of 30 msec, a total number of 180° cycles 2, 256 transients, a spectral width of 7.2 kHz, 32,768 (32K) data points, and a 3-sec TR. The choice of a spin-echo delay of 30 msec, was based on the observation that at this echo-time we avoided line broadening without loss of signals from triglycerides. When we increased the spin-echo delay, this affected all lipid signals but not in favor of other metabolites.

We also performed 1D water presaturation Nuclear Overhauser Effect SpectroscopY (NOESY) (36,37). Acquisition parameters were: mixing time (τ_m_=70 and 100 msec), relaxation delay of 3 sec, 32 scans, 16 dummy scans, 32,768 (32K) data points.

Two-dimensional (2D)^1^H-^1^H HRMAS MRS single-fly spectra were acquired on all samples using a TOBSY sequence with adiabatic pulses (16). Acquisition parameters were: 2K data points direct dimension (11 ppm spectral width), 1-sec water pre-saturation during the relaxation delay, 8 scans per increment, 128 increments, 2-sec total repetition time, 45-msec mixing time, and a total acquisition time of 29 min. 2D ^1^H, ^13^C-heteronuclear single quantum coherence (HSQC) (38) spectra were acquired using an echo-time phase sensitive standard pulse sequence (hsqcedetgp) and 0.5-sec relaxation delay, 1.725 msec evolution time, 2 kHz spectra width in f2, 2K data point (Time Domain, TD), 128 scans for increment, 17 kHz spectra width in f1, 256 increments, heteronuclear scalar J (^13^C, ^1^H) coupling 145 Hz (CNST2), presaturation of water resonance, in combination with gradient selection, to suppress the water signal; total acquisition time 16 h.

### In vivo ^1^H HRMAS MRS data processing

MR spectra of specimens were analyzed using MestReC software (Mestrelab Research, www.mestrec.com). A 0.5-Hz line-broadening apodization function was applied to CPMG HRMAS ^1^H FIDs prior to Fourier transformation (FT). MR spectra were referenced with respect to TSP at δ=0.0 ppm (external standard), manually phased, and a Whittaker baseline estimator was applied to subtract the broad components of the baseline.

The parameters for processing the 2D TOBSY MR spectra were: QSINE=2 window function in both dimensions, FT with 2K points in the direct dimension and zero-filling to 1K in the second dimension, phase correction in both dimensions and baseline correction in the second dimension. The parameters for processing the 2D HSQC MR spectra were: QSINE=2 window function in both dimensions, FT with 2K points in the direct dimension and zero-filling to 512 in the second dimension, phase correction in both dimensions. Processing of all 2D MR spectra was completed using XWINNMR 3.5 software (Bruker Bruker Biospin Corp., Billerica, MA). To quantify and illustrate the 2D NMR spectra we used the Sparky program (T.D. Goddard and D.G. Kneller, SPARKY 3, USCF, http://www.cgl.ucsf.edu/home/sparky/).

### Quantification of metabolites from 1D CPMG spectra

For metabolite quantification, we used the ‘external standard’ technique, which provides highly accurate values. For the quantification, we used the 1D ^1^H CPMG HRMAS spectra. Metabolite concentrations were calculated using the MestReC software (Mestrelab Research, www.mestrec.com). An automated fitting routine based on the Levenberg-Marquardt algorithm (39,40) was applied after manual peak selection; peak positions, intensities, linewidths and Lorentzian/Gaussian ratios were adjusted until the residual spectrum was minimized. Metabolite concentration (mol/kg) was calculated using the following equation (41):

$${\text{mass}}_{\text{TSP}}/{\text{PM}}_{\text{TSP}}*{\text{Met}}_{\left(\text{area}\right)}/{\text{TSP}}_{\left(\text{area}\right)}*{\text{N}}_{\text{TSP}/}{\text{N}}_{\text{Met}}*1/\mathrm{wt}(\text{sample})$$

where, mass_TSP_ was constant (0.069 mg), PM_TSP_ was the molecular weight of TSP (172.23 g/mol), Met signifies metabolites, N_TSP_ was the TSP proton number (9 ^1^H), N_Met_ was the metabolite proton number, and *wt* was the sample weight in mg.

### Quantification of metabolites from 2D TOBSY spectra

To quantify more metabolites, we used the ratio of the Cross Peak Volume of the Metabolites [CVP(M)] to the TSP Diagonal Peak Volume [DPV/TSP] as described previously (14). This ratio was further divided by sample weight (*wt)* to yield normalized metabolite intensity, Ic(M) = (1/*wt*) * CPV(M)/DPV(TSP).

### Statistics

Statistical comparison was done using ANOVA with the Bonferroni correction to account for multiple of comparisons. A P-value of 0.05 (corrected) was used for significance and P-values are reported with two significant digits. Calculations were performed using SPSS (SPSS 12, SPSS Inc.).

## Results

**Figure 2:**
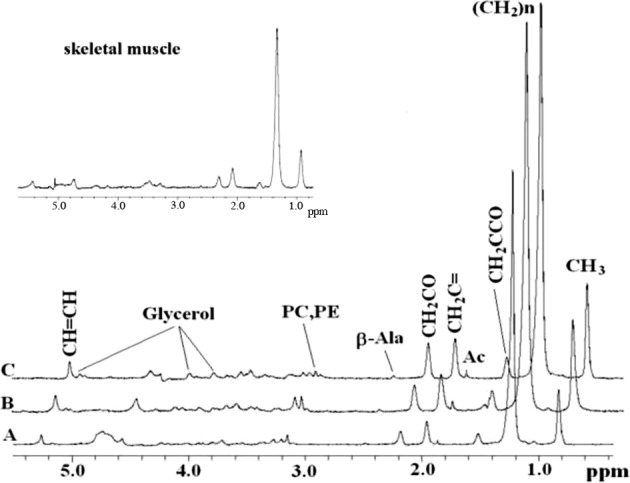
*In vivo* 1D HRMAS ^1^H CPMG spectra of: (A) young *wt* injured, (B) old *wt* injured, and (C) young *imd* injured flies. Lipid components: CH_3_ (0.89 ppm), (CH_2_)n (1.33 ppm), CH_2_C-CO (1.58 ppm), acetate (Ac, 1.92 ppm), CH_2_C═C (2.02 ppm), CH_2_C═O (2.24 ppm), ß-alanine (ß-Ala, 2.55 ppm), phosphocholine (PC, 3.22 ppm), and phosphoethanolamine (PE, 3.22 ppm) glycerol (4.10, 4.30 ppm 1,3-CH; 5.22 ppm 2-CH_2_), CH═CH (5.33 ppm). The spectra in the insert are from the thorax of dissected flies and thus represent primarily skeletal muscle; note their similarity to spectra for whole flies. Shown spectra were normalized to TSP at each echo time and therefore do not exhibit T_2_ decay.

Fig. 2 presents 1D ^1^H HRMAS CPMG spectra from young and aged *wt* flies as well as young *imd* flies that had been injured. Also shown (insert) is an 1D ^1^H HRMAS CPMG summed spectrum from the thorax of dissected flies; this spectrum represents primarily skeletal muscle because fly thorax is highly enriched in skeletal muscle and is similar to the spectra from whole flies (rest of the spectra shown herein). Principal lipid components [CH_3_ (0.89 ppm), (CH_2_)n (1.33 ppm), CH_2_CCO (1.58 ppm), CH_2_C═C (2.02 ppm), CH_2_C═O (2.24 ppm), CH═CH (5.33 ppm)], glycerol (4.10, 4.30 and 5.24 ppm), acetate (Ac, 1.92 ppm), ß-alanine (ß-Ala, 2.55 ppm), phosphocholine (PC, 3.22 ppm), and phosphoethanolamine (PE, 3.22 ppm) were detected in accordance with prior reports (11,42). Signals at 2.02 ppm were assigned to methylene protons of the CH_2_-CH═CH moiety of monounsaturated fatty acids (i.e. palmitoleic). Interestingly, we did not detect poly-unsaturated fatty acids (PUFAs), and thus the signal at 2.78 ppm, attributable to the methylene protons between two double bonds (═C-CH_2_-C═) in poly-unsaturated acids, was not present. However, PUFAs were detectable in female flies (unpublished data). The unsaturated acids were identified by a signal at 5.33 ppm produced by protons of the -CH═CH-moiety.

In the NOESY experiments (data not shown) when we increased the mixing time (from 70 msec to 100 msec) the lipids components decreased but not in favor of small metabolites, in other words the lipid signals were attenuated, but this signal reduction was not in favor of smaller metabolites. Thus, the NOESY and CPMG findings were similar to each other.

In Table I, we report the chemical shifts obtained from 1D ^1^H CPMG MR spectra and the quantities of lipids components that characterized the flies in our study. Most lipid resonances were significantly elevated. Note that apart from the 1.33 ppm and other lipids, the ceramide derived olefinic protons (CH═CH at 5.33 ppm) were significantly increased after injury in *wt* old and *akhr* flies (Table I). Injury did not significantly affect the metabolite profile of young *wt* flies (Table I). Injury, however, did affect the metabolic profile of aged *wt* flies was similar to the profile of old *imd* flies (Table I).

We measured the T2 of metabolites and TSP from 1D ^1^H CPMG spectra at different echo times (TE at 30, 60, 100, 300, 450 and 600 msec). Our results showed that the T2 decay rate of TSP (1,125±103 msec) is almost identical to that of CH_3_ group at 0.89 ppm (1,156±72 msec); moreover, the T2s of (CH_2_)n at 1.33 ppm (516±14 msec), CH_2_C═C at 2.02 ppm (537±35 msec) and CH═CH at 5.33 ppm (469±27 msec) were almost identical to each other and half of the T2 of TSP and CH_3_, CH_2_CCO at 1.58 ppm (292±5 msec) and CH_2_CO at 2.24 ppm (265±16 msec). Even at an echo time of 600 msec, these peaks would not have totally decayed, meaning that TSP and lipid do not relax differently.

**Figure 3:**
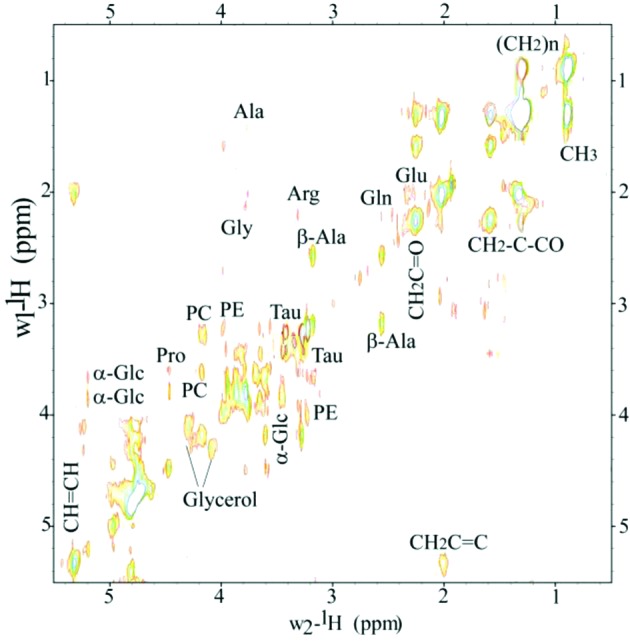
Representative 2D ^1^H-^1^H TOBSY HRMAS spectrum of live *Drosophila* at 14.1 T. Small metabolites and lipid components were identified. Metabolites: alanine (Ala), ß-alanine (ß-Ala), arginine (Arg), glutamine (Gln), glutamate (Glu), phosphocholine (PC), phosphoethanolamine (PE), Taurine (Tau), α-glucose (α-Glc) and glycerol. Lipids components: CH_3_ (0.89 ppm), (CH_2_)n (1.33 ppm), CH_2_C-CO (1.58 ppm), CH_2_C═C (2.02 ppm), CH_2_C═O (2.24 ppm), CH═CH (5.33 ppm).

**Figure 4:**
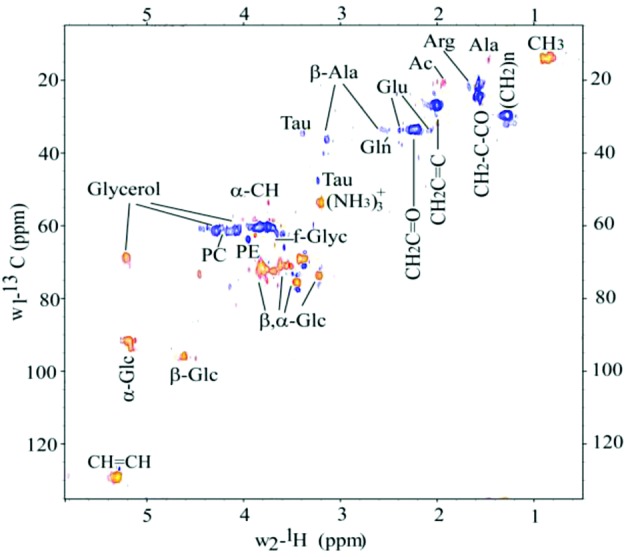
Representative ^1^H, ^13^C HSQC spectrum of live *Drosophila* at 14.1 T. We identified small metabolites: alanine (Ala), ß-alanine (ß-Ala), arginine (Arg), glutamine (Gln), glutamate (Glu), PC phosphocholine (PC), phosphoethanolamine (PE), Taurine (Tau), ß, α-Glucose (ß, α-Glc), free glycerol (f-Glyc) and bonded glycerol (Glycerol); and lipid components: CH_3_, (CH_2_)n, CH_2_C-CO, CH_2_C═C, CH_2_C═O, CH═CH. Note the HSQC acquisition was performed using 10 wild-type flies (weight 9.4 mg); the acquisition time for this experiment was ∼16 h.

Metabolites that could not be assigned or were not visible using the 1D spectrum were detected using selected 2D experiments such as 2D TOBSY (Fig. 3), and HSQC (Fig. 4); and their assignment was confirmed by comparison with literature data. HSQC spectra revealed directly bonded carbon-proton pairs, thus enabling the assignment of singlets (which do not give correlations in homonuclear TOBSY spectra), and the discrimination among compounds having similar protons but diverse ^13^C chemical shifts. The experiments provided complete and unambiguous identification of the metabolic pattern characterizing *Drosophila*. The main mobile lipids and small metabolites are reported in Table II.

**Figure 5:**
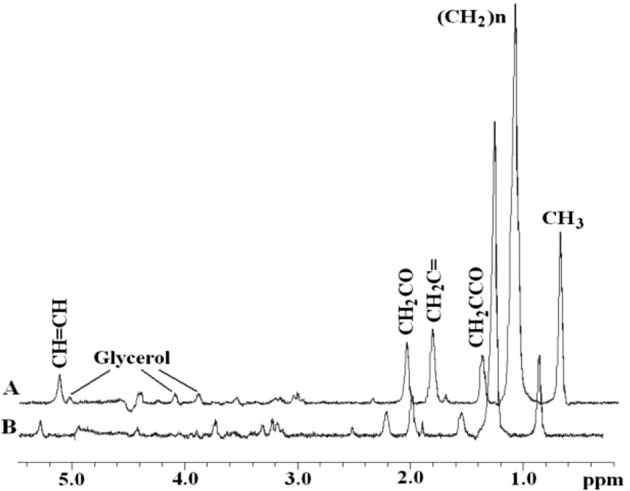
*In vivo* 1D HRMAS ^1^H CPMG spectra of (A) adipokinetic hormone receptor mutant *Drosophila akhr^null^*, and the isogenic control *akhr^rev^* (B). The (CH_2_)n lipids at 1.33 ppm and the CH_2_C-CO lipids at 1.58 ppm attributed to both IMCLs and EMCLs were increased in the *akhr^null^* mutant. Note: Shown spectra were normalized to TSP at each echo time and therefore do not exhibit T_2_ decay.

**Figure 6:**
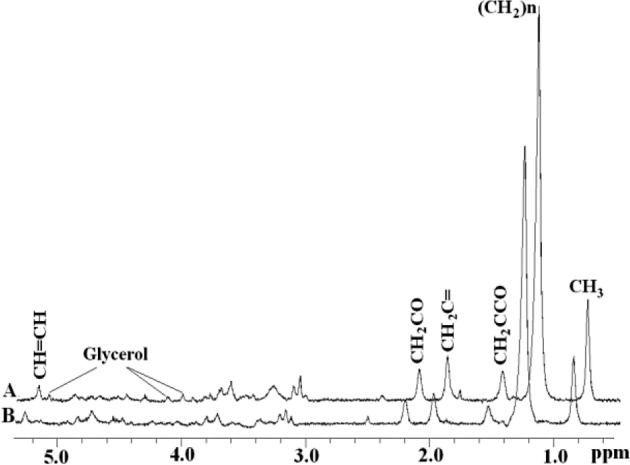
*In vivo* 1D HRMAS ^1^H CPMG spectra of (A) flies mutated at the *chico* gene, a *Drosophila* homolog of vertebrate insulin receptor substrate 1–4 (IRS1–4), and the isogenic control strain flies (B). Note: Shown spectra were normalized to TSP at each echo time and therefore do not exhibit T_2_ decay.

Representative *in vivo* 1D HRMAS ^1^H CPMG spectra of *akhr^null^* mutant *Drosophila* and its isogenic control *akhr^rev^* are shown in Fig. 5. Note that the metabolic profile of the *akhr^null^* mutant, which has a phenotype of obesity, showed a substantial increase in both (CH_2_)n lipids at 1.33 ppm and CH_2_C-CO lipids at 1.58 ppm, as well as increases in other lipids (Table I). The *akhr^null^* mutant flies also showed an increase in the amount of bonded glycerol (signals at 4.10, 4.30 and 5.24 ppm), with respect to the control *akhr^rev^* flies. On the other hand, *chico* flies which are mutated at the insulin signaling pathway exhibited significantly increased lipid peaks at 0.89 ppm (CH_3_), at 1.33 ppm (CH_2_)_n_ and also at 2.02 ppm (CH_2_C═) with respect to the genetic control (Fig. 6, Table I).

## Discussion

In the present study, we demonstrate the implementation of a novel *in vivo* HRMAS ^1^H NMR approach for detecting biologically important molecules. Specifically, we detected lipids and small metabolites in live *Drosophila* at 14.1 T in ∼45 min. Our results confirmed our expectations in that we were able to reduce acquisition time, thus achieving zero mortality. We introduced a novel *in vivo* HRMAS ^1^H-MRS approach in *Drosophila* which we used to test the hypothesis that trauma and innate immunity are linked to reduced insulin signaling, a phylogenetically conserved pathway for regulation of glucose and lipid metabolism (27,28).

The use of a rotor-synchronized WURST-8 adiabatic pulse (C^9^_1_15) permitted us to obtain a satisfactory SNR and good resolution of tissue spectra relative to the use of an isotropic mixing pulse (MLEV-16), in agreement with previous studies (16,43). Our ability to use TOBSY to detect an improved metabolic profile of *Drosophila* suggests that TOBSY used with 1D CPMG is well suited for simultaneous qualitative and quantitative analysis of metabolite concentrations and enables improved evaluation of metabolic dysfunction in *Drosophila*.

Our *in vivo* fly spectra compare well to other published *in vivo* skeletal muscle spectra (11,44,45). All of these works show high amounts of lipids (in particular triglycerides). Other HRMAS reports on skeletal muscle show spectra with more metabolites (8,46). In our case, the samples and set conditions in our experiments were different, we used a small amount of sample (between 0.6 and 1.1 mg) and performed the experiment with a lower spin rate, which may have an effect on spectral resolution. Since a single *Drosophila* fly weighs ∼0.7–0.8 mg total body weight, the NMR-visible non-lipid components are expected to contribute only a small percentage to the total signal with concomitantly little sensitivity of detection. Even spectra from the thorax of dissected flies representing primarily skeletal muscle since fly thorax is highly enriched in skeletal muscle are similar to the spectra from whole flies (insert of Fig. 1). However, as shown we were able to detect certain metabolites from the 1D experiment (Fig. 2) and then we improved and confirmed our results using the 2D TOBSY experiment (Fig. 3).

From a biomedical perspective, a principal finding of our experiments was that mobile lipids accumulated in muscle tissue in response to injury (Fig. 2). Although determining the source of these accumulated lipids is beyond the scope of this study, it has previously been shown that EMCLs, IMCLs, and triglycerides can all contribute to cellular lipid peaks (19,47,48). Indeed, EMCLs and IMCLs can be distinguished by *in vivo* MRS due to differences in bulk magnetic susceptibility and geometric arrangements (49) and 1.33-ppm lipids have been attributed to IMCLs whereas 1.58-ppm lipids have been attributed to EMCLs. However, in our study this discrimination may not be possible. Spinning a sample at the magic angle (HRMAS) with respect to the static field direction averages the second-order tensors of the anisotropic chemical shift, the dipolar interaction, and the susceptibility variations in heterogeneous samples (50–52). Garroway (51) indicated that MAS not only eliminates the broadening effect due to magnetic susceptibility but also the shift itself. Later, Chen *et al*(53) clarified that irrespective of the system geometry, MAS removes only the anisotropic contribution of bulk susceptibility inside an homogeneous susceptibility region. Inspecting the isotropic part of the susceptibility tensors available for IMCLs and EMCLs (47,54) we can deduce that under MAS conditions IMCLs and EMCLs have the same chemical shift due to bulk susceptibility.

IMCLs probably serve as an energy substrate for oxidative metabolism (55), and can be mobilized and utilized with a turnover times of several hours (56). In insects, triglycerides are located in body fat (57–59) and are used both for energy storage and for storage of fatty acid precursors, such as transported lipids, phospholipids (membrane structure), hydrocarbons, and wax esters (minimize water loss from the cuticle due to evaporation) (60). In our study, mobility of fat body contents may have been affected by trauma or immune status, thus giving rise to increased IMCL and EMCL signals (61). However, this is only speculation as the intracellular signaling cascade mediating mobilization of triglycerides has not been as fully elucidated in insects as it has in mammals (30). Nevertheless, we propose that there was mobilization of triglycerides in the *akhr* flies because the peaks indicative of triglycerides at 1.33 ppm and 1.58 ppm were increased (Table I). The significant increase in triglycerides (both due to IMCLs and EMCLs) detected in the *akhr^null^* mutants is in agreement with their obese phenotype and abnormal accumulation of both lipids and carbohydrates (62,63). Indeed, elevated IMCL levels are associated with insulin resistance, a major metabolic dysfunction of diabetes (64,65), aging (66,67), burn trauma (68–70) and obesity (71).

Previous measurements of muscle triglyceride content by biopsy and IMCL content by ^1^H NMR spectroscopy have shown a strong relationship between intramuscular fat content and insulin resistance in muscle. While increased fatty acid delivery from lipolysis could also produce the observed IMCL increase, free fatty acid concentrations may be highly variable in traumatized patients (72). Also, impaired lipoprotein and PUFA metabolism occurs in the early post-trauma period, implicating their involvement in subsequent healing and immune function. The presently observed IMCL increase, however, was not accompanied by evidence of detectable PUFAs in our experiments. According to Chertemps *et al*(73), however, elongase involved in the hydrocarbon biosynthesis of sex pheromones (which is a long-chain hydrocarbon but shorter in females by one double carbon bond) may be absent or present in very low amounts in male flies. Thus, the absence of PUFA in our data may be related to our use of male flies. Previous genomic (74) and gene expression data in human diabetes (75) suggest that increased IMCL levels could be the result of decreased mitochondrial oxidative capacity. Increased IMCL levels have also been reported to be associated with insulin resistance in type 2 diabetes, suggesting reduced mitochondrial oxidation and phosphorylation.

Interestingly, we observed a marked increase in the same peaks at 1.33 ppm and 1.58 ppm in injured, aged *wt* flies, which can also be attributed to mobilization of triglycerides. Thus, metabolism of body fat in the aged injured flies may be similar to that in *akhr* obese phenotype flies (31). These observations suggest that *Drosophila* could be a useful model not only for studying aging but also obesity. Nevertheless, they do not clearly indicate whether the increase of triglycerides is attributed to insulin resistance, which is not only associated with obesity (76,77) but also with trauma.

On the other hand, our observations of significantly increased peaks indicative of triglycerides at 1.33 ppm in *chico* flies (Table I) suggest that *Drosophila* could be also a useful model for studying insulin signaling since these flies with mutation in insulin receptor substrate (IRS), a *Drosophila* homolog of vertebrate IRS1-4, indeed show substantial increase in triglycerides (32,78) due to a mutated insulin signaling pathway (27), which causes reduced signaling through this pathway and insulin resistance. Clearly, in the *chico* flies the increase at 1.33-ppm peak is due to IMCLs and not due to EMCLs since these flies are not reported to be obese. Interestingly, the *chico* flies do not exhibit significantly increased 1.58-ppm peaks which are frequently attributed to EMCLs. It is anticipated that the *chico* flies should not have increased EMCLs since they are dwarf flies and not obese. Thus, it may be, in spite of the theoretical considerations of HRMAS, that the lipids that give rise to the peak at 1.33 ppm are due primarily to IMCLs whereas the lipids that give rise to the peak at 1.58 ppm are primarily due to EMCLs. In any case, the *chico* flies are the proper control for the aged-traumatized and immune-deficient flies, which also exhibit increased triglycerides, evidently due to increased IMCLs and not due to EMCLs since they are not obese, and thus not expected to have increased EMCLs. The aged traumatized and immune-deficient flies show a very similar metabolic profile to the *chico* flies by exhibiting significantly increased lipids at 0.89 and 1.33 ppm, which suggests derangements in the insulin signaling pathway and possibly insulin resistance observed in mammals. On the other hand, the *akhr* flies exhibit a metabolic profile with significantly increased peaks in all assigned lipids, which agrees with their obese phenotype.

Another principal finding of our experiments was that ceramide accumulated in aged injured, or obese flies (Table I and Fig. 3). Ceramide accumulation decreases insulin stimulated GLUT4 translocation to the plasma membrane and, consequently, decreases glucose transport (79), resulting in insulin resistance. Honjo and co-workers demonstrated that saturated fatty acids (such as palmitoleic acid, signal at 2.02 ppm in our study) induce *de novo* synthesis of ceramide and programmed cell death (79). They suggested that inhibition of carnitine palmitoyltransferase I activity induced both sphingolipid synthesis and palmitate-induced cell death. Meanwhile, Ruddock *et al*(80) suggested that long chain saturated fatty acids (palmitoleic acid C16:0) inhibit insulin action and attenuate insulin signal transduction in hepatoma cell lines. Their work suggests that an increase in palmitoleic acid signifies insulin resistance. If so, the signal at 2.02 ppm in our study may also be a biomarker of insulin resistance and this peak was increased in aged *imd, akhr* and *chico* flies (Table I).

Finally, from a biomedical perspective, the findings of this study support the hypothesis that trauma and innate immunity are linked to insulin signaling and suggest that IMCL may be a biomarker of insulin resistance in injury, aging, obesity and immuno-deficiency. Insulin resistance has been suggested to develop following critical illness and severe injury (76). Whether IMCL is an instigator or a marker of insulin resistance is currently a topic of debate (81). Insulin resistance has not been previously demonstrated in flies using currently available assays. Furthermore, direct links between innate immune deficiency and signaling which lead to insulin resistance in mammals, as suggested in this study, have not been made previously, with the exception of a recent study of biological data in *Drosophila* that confirm our HRMAS findings (82). The common characteristics shared among innate immunity activation, obesity, and insulin resistance, as recently described, also support the findings of this study.

In conclusion, we demonstrated that a novel solid-state HRMAS TOBSY NMR method is a sensitive tool in the molecular characterization of metabolic perturbations in *Drosophila*. We observed increased levels of triglycerides in injury, innate immunity, aging and obesity that may be indicative of insulin resistance. These findings may thus be directly relevant to the mitochondrial dysfunction and muscle wasting that occur in trauma, aging and immune system deficiencies that lead to heightened susceptibility in infection. Our approach advances the development of novel *in vivo* non-destructive research approaches in *Drosophila*, offers biomarkers to investigate biomedical paradigms, and thus may direct novel therapeutic development.

## Figures and Tables

**Table I:** Chemical shift and quantity (*μ*mol/g) of selected lipid components in live *Drosophila* from 1D CPMG measurements.

		Lipid components					
		CH_3_	(CH_2_)n	CH_2_CCO	CH_2_C═	CH_2_CO	CH═CH
				Chemical shift (δ, ppm)			

		0.89 ppm	1.33 ppm	1.58 ppm	2.02 ppm	2.24 ppm	5.33 ppm
*wt*							
Young	Not injured	0.14±0.01	1.17±0.10	0.050±0.008	0.13±0.01	0.090±0.009	0.07±0.02
	Injured	0.18±0.02	1.50±0.14	0.08±0.01	0.16±0.02	0.12±0.01	0.09±0.01
	% change	28.57	28.21	60.00	23.08	33.33	28.57
	P-value	0.30	0.080	0.33	0.19	0.26	0.64
Old	Not injured	0.18±0.01	1.41±0.08	0.060±0.003	0.13±0.01	0.07±0.01	0.08±0.01
	Injured	0.27±0.03	2.10±0.25	0.16±0.08	0.24±0.06	0.13±0.03	0.13±0.02
	% change	50.0	48.94	166.67	84.62	85.71	62.50
	P-value	0.022^a^	0.024^a^	0.26	0.085	0.071	0.015^a^
*imd*							
Young	Not injured	0.34±0.02	2.48±0.19	0.13±0.02	0.26±0.02	0.21±0.02	0.17±0.01
	Injured	0.38±0.04	2.56±0.26	0.15±0.01	0.27±0.03	0.22±0.02	0.19±0.02
	% change	11.76	3.23	15.38	3.85	4.76	11.76
	P-value	0.38	0.80	0.52	0.81	0.88	0.40
Old	Not injured	0.27±0.03	1.71±0.19	0.11±0.02	0.20±0.03	0.15±0.02	0.11±0.02
	Injured	0.36±0.02	2.83±0.61	0.11±0.01	0.28±0.02	0.16±0.02	0.16±0.02
	% change	33.33	65.50	0.00	40.00	6.67	45.45
	P-value	0.048^a^	0.0050^a^	0.74	0.044^a^	0.68	0.12
*akhr*	Isogenic control	0.13±0.02	1.01±0.13	0.05±0.01	0.11±0.01	0.06±0.01	0.06±0.01
	Knockout	0.35±0.05	2.67±0.38	0.14±0.02	0.26±0.04	0.19±0.03	0.12±0.01
	% change	169.23	164.36	180.00	136.36	216.67	100.00
	P-value	0.0015^a^	0.0011^a^	0.0013^a^	0.0013^a^	0.00019^a^	0.0020^a^
*chico*	Control	0.20±0.03	1.30±0.14	0.06±0.02	0.15±0.04	0.10±0.02	0.08±0.02
	Chico null	0.37±0.03	2.09±0.14	0.07±0.01	0.29±0.05	0.17±0.03	0.12±0.01
	% change	17.43	78.54	1.62	14.30	7.16	3.42
	P-value	0.0021^a^	0.0024^a^	0.53	0.046^a^	0.077	0.10

Values are expressed as means ± standard errors (SE); % change = percent change; P-values were calculated using ANOVA with the Bonferroni correction to account for multiple comparisons;

a statistical significance.

**Table II:** Small metabolites and lipid components identified using 2D TOBSY from *Drosophila*.

Metabolites	δ^1^H (ppm)	δ^13^C (ppm)	Group
Lipids components	0.89	14.6	CH_3_
	1.33	29.7	(CH_2_)n
	1.58	24.8	CH_2_C-C═O
	2.02	27.0	CH_2_C═
	2.24	33.5	CH_2_C═O
	5.33	129.1	CH═CH
Acetate	1.98	24.6	CH_3_
Alanine	1.48	16.1	CH_3_
	3.78	55.1	CH
ß-alanine	2.55	33.43	CH_2_
	3.16	36.1	CH_2_
Arginine	1.64	24.4	γ-CH_2_
	3.26	41.0	δ-CH_2_
Glutamate	2.09	27.9	ß-CH_2_
	2.35	33.7	γ-CH_2_
	3.78	55.1	α-CH_2_
Glutamine	2.17		ß-CH_2_
	2.44	31.5	γ-CH_2_
	3.78	55.1	α-CH
Free glycerol	3.56, 3.65	63.3	1,3-CH_2_
Glycerol	4.10, 4.30	61.7	1,3-CH_2_
	5.24	69.8	CH
Glycine	3.55		CH_2_
α-glucose	5.22	92.6	1-CH
	3.59	72.0	2-CH
	3.88	72.5	5-CH
ß-glucose	4.67	96.6	1-CH
	3.26	74.8	2-CH
	3.48	76.6	3-CH
Phosphoethanolamine	3.98	63.8	N-CH_2_
	3.20		O-CH_2_
Phosphocholine	4.17	61.7	O-CH_2_
	3.59		N-CH_2_
	3.22	53.7	N(CH_3_)_3_
Proline	2.10		ß'-CH
	3.31		γ-CH_2_
	4.14		α-CH_2_
Taurine	3.26	48.0	S-CH_2_
	3.42	34.6	N-CH_2_

## Abbreviations:

Ac: acetate
*akhr*: adipokinetic hormone receptor
Ala: alanine
ß-Ala: ß-alanine
Arg: arginine
CPMG: Carr-Purcell-Meiboom-Gill
EMCLs: extramyocellular lipids
FFA: free fatty acids
α-Glc, ß-Glc: α-, ß-glucose
Glu: glutamate
Gln: glutamine
HRMAS: high-resolution magic angle spinning
*imd*: immune deficiency
IMCLs: intramyocellular lipids
HSQC: heteronuclear single quantum coherence
Lip: lipids
PE: phosphoethanolamine
PC: phosphocholine
Pro: proline
PUFA: poly-unsaturated fatty acid
Tau: taurine
TOBSY: TOtal through Bond correlation SpectroscopY
TOCSY: TOtal Correlation SpectroscopY
TGA: triglycerides
*wt*: wild-type
